# Interference with distinct steps of sphingolipid synthesis and signaling attenuates proliferation of U87MG glioma cells

**DOI:** 10.1016/j.bcp.2015.05.007

**Published:** 2015-07-15

**Authors:** Eva Bernhart, Sabine Damm, Andrea Wintersperger, Christoph Nusshold, Anna Martina Brunner, Ioanna Plastira, Gerald Rechberger, Helga Reicher, Christian Wadsack, Andreas Zimmer, Ernst Malle, Wolfgang Sattler

**Affiliations:** a Institute of Molecular Biology and Biochemistry, Medical University of Graz, Austria; b BioTechMed Graz, Austria; c Institute of Molecular Biosciences, University of Graz, Austria; d Department of Obstetrics and Gynecology, Medical University of Graz, Austria; e Institute of Pharmaceutical Sciences, Department of Pharmaceutical Technology, University of Graz, Austria

**Keywords:** ABC transporter, Myriocin, p53, Proliferation, RNA interference, Sphingolipids

## Abstract

Glioblastoma is the most common malignant brain tumor, which, despite combined radio- and chemotherapy, recurs and is invariably fatal for affected patients. Members of the sphingolipid (SL) family are potent effectors of glioma cell proliferation. In particular sphingosine-1-phosphate (S1P) and the corresponding G protein-coupled S1P receptors transmit proliferative signals to glioma cells. To investigate the contribution to glioma cell proliferation we inhibited the first step of de novo SL synthesis in p53^wt^ and p53^mut^ glioma cells, and interfered with S1P signaling specifically in p53^wt^ U87MG cells. Subunit silencing (RNAi) or pharmacological antagonism (using myriocin) of serine palmitoyltransferase (SPT; catalyzing the first committed step of SL biosynthesis) reduced proliferation of p53^wt^ but not p53^mut^ GBM cells. In U87MG cells these observations were accompanied by decreased ceramide, sphingomyelin, and S1P content. Inhibition of SPT upregulated p53 and p21 expression and induced an increase in early and late apoptotic U87MG cells. Exogenously added S1P (complexed to physiological carriers) increased U87MG proliferation. In line, silencing of individual members of the S1P receptor family decreased U87MG proliferation. Silencing and pharmacological inhibition of the ATP-dependent cassette transporter A1 (ABCA1) that facilitates S1P efflux in astrocytes attenuated U87MG growth. Glyburide-mediated inhibition of ABCA1 resulted in intracellular accumulation of S1P raising the possibility that ABCA1 promotes S1P efflux in U87MG glioma cells thereby contributing to inside-out signaling. Our findings indicate that de novo SL synthesis, S1P receptor-mediated signaling, and ABCA1-mediated S1P efflux could provide pharmacological targets to interfere with glioma cell proliferation.

## Graphical abstract

**Figure:**
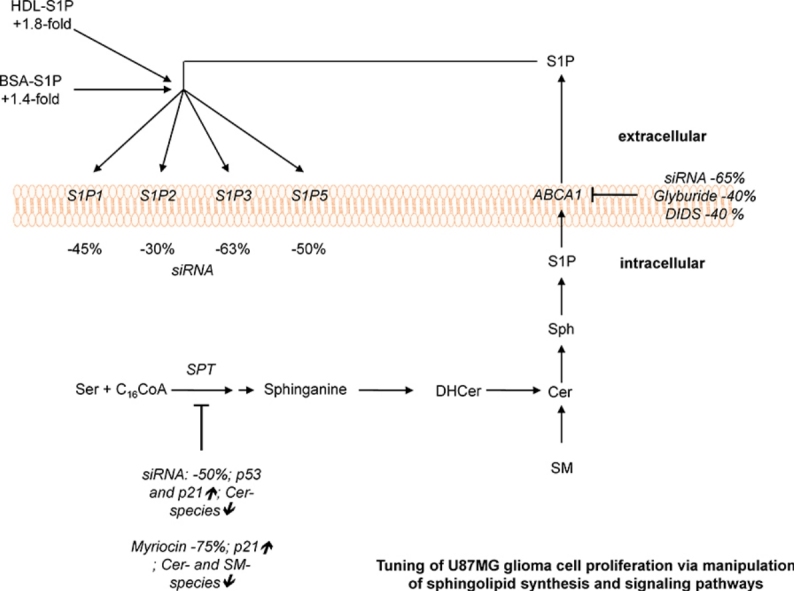

## 1 Introduction

Glioblastoma (GBM; astrocytoma grade IV) tumors are the most common type of primary brain tumors occurring in adult patients. The effectiveness of treatments is limited due to the high proliferative potential and the diffusely infiltrating properties of the tumor [1,2].

Sphingolipid (SL) metabolites represent a major class of bioactive lipids that regulate a plethora of cellular functions, including proliferation, differentiation, migration, and apoptosis [3]. Therefore it is not surprising that dysregulated SL metabolism contributes to cancer progression and could provide a pharmacological target to develop new chemotherapeutics [4]. The central metabolite of SL turnover is ceramide (Cer). In the first rate-limiting step of de novo synthesis serine palmitoyltransferase (SPT) catalyzes the condensation of serine and palmitoyl-CoA and a series of subsequent reactions including Cer synthases (CerS) generate Cer [3,5]. Alternatively Cer can be generated by hydrolysis of sphingomyelin (SM) via the action of sphingomyelinases (SMases) or from glycosphingolipids.

Members of the CerS family catalyze the formation of Cer from sphingosine and acyl-CoA substrates. This family of enzymes takes a unique role in SL metabolism in that they regulate de novo SL synthesis and the recycling of free sphingosine from degradation of the endogenous SL pool via the Salvage pathway [6]. Each of the six CerS is able to synthesize Cer species with characteristic acyl-chain lengths [7]. De-acylation of Cer yields sphingosine, which can be phosphorylated (via sphingosine kinase 1 or 2; SK1/2) to yield sphingosine-1-phosphate (S1P). Thus, Cer, sphingosine, and S1P are readily interconvertible resulting in a highly dynamic SL pool. This is of importance since the ‘balance’ of this SL rheostat determines cell fate [7]. Cer typically induces growth arrest and/or apoptosis in response to stress signals while S1P inhibits apoptosis and induces cell proliferation [8]. Therefore, tuning of the SL rheostat in favor of S1P results in a cellular survival benefit for tumor cells whereas Cer generation inhibits tumorigenesis [4].

S1P-mediated signaling is elicited by five G protein-coupled receptors termed S1P_1–5_. By activation of specific downstream effector molecules, these receptors induce a variety of cellular responses many of them central to tumor biology [8] including cell transformation, survival, migration, metastasis, and angiogenesis [3,8–11]. Accumulating evidence suggests that S1P, SK, and S1P receptors are central players that regulate GBM growth, migration, and invasion via outside-in or inside-out signaling [12]. Exogenously added S1P is a potent glioblastoma mitogen and enhances glioblastoma invasiveness [13–17]. Microarray analyses suggest that upregulation of proteases in response to exogenous S1P could be key to invasive properties of glioblastoma cells [18]. Only recently a systematic shift in SL metabolism favoring S1P over Cer generation in GBM was demonstrated [19]. Furthermore inhibition of S1P production in GBM cells resulted in decreased angiogenesis of co-cultured endothelial cells [19].

S1P receptors are expressed in GBM tissues and cell lines [20,21]. Overexpression of S1P_1_ correlates with high invasive potential of CD133^+^ GBM cells [15,16]. S1P_2_ inhibits GBM cell migration [22–24] but increases invasive potential [24]. SK1 is upregulated in GBM and expression levels are linked to reduced survival [20]. Concomitantly it was shown that interleukin-1-mediated upregulation of SK1 increases growth rates and invasiveness of GBM cells [25]. Accordingly, pharmacological inhibition of SK induces apoptosis of GBM cells in vitro [26], reduces GBM xenograft growth in vivo [27], and increases the anti-proliferative potential of temozolomide in glioma cell cultures [28].

Many cell types are able to secrete S1P and evidence suggests that ATP-binding cassette (ABC) transporters are involved in this pathway. S1P release from mast cells and platelets is mediated by ABC transporters [29,30]. In astrocytes ABCA1 is responsible for S1P export [31]. Pharmacological compounds that shift the SL pattern toward a more anti-proliferative phenotype could be suitable co-adjuvants in combination with common chemotherapeutics [32]. Therefore the present in vitro study aimed at investigating the contribution of the committed step of de novo SL biosynthesis, individual members of the S1P receptor family, and the putative S1P efflux pump ABCA1 to GBM cell proliferation.

## 2 Materials and methods

### 2.1 Materials

Cell culture supplies were from Gibco (Invitrogen, Vienna, Austria), PAA Laboratories (Linz, Austria) and Costar (Vienna). 4,4′-Diisothiocyanostilbene-2,2′-disulfonic acid (DIDS), SuperScript III Reverse Transcriptase and Oligofectamine were from Invitrogen (Vienna). Random Hexamer Primer was from Thermo Scientific (MA, USA). Myriocin, 25-hydroxycholesterol (25-OHcholesterol), glyburide, bovine serum albumin (BSA), monoclonal anti-β-actin, and horseradish peroxidase (HRP)-labeled goat anti-rabbit IgG were from Sigma (Vienna, Austria). 24-OHcholesterol was from Steraloids (Newport, CT). TO901317 was from Cayman Europe (Tallin, Estonia). S1P, anti-S1P_2_ (H-64), anti-S1P_5_ (H-88), anti-p21 (187), anti-SPTLC1 (H-300; a catalytic subunit of SPT), and HRP-labeled goat anti-mouse IgG were from Santa Cruz (Santa Cruz Biotechnology, CA, USA). Anti-S1P_1_ (AB-236) was from Assay Bio Tech (Antibodies-Online, Aachen, Germany), anti-S1P_3_ (ab74477) was from Abcam (Cambridge, UK), and anti-p53 (clone DO-7) was from DakoCytomation (Gostrup, DK). SuperSignal Western blot detection reagent kit was from Pierce (Thermo Scientific, MA, USA) and ECL Plus Western Blotting Reagents were form Amersham Biosciences (Vienna). RNeasy Plus Kit, QuantiFast SYBR Green PCR kit, QuantiTect primer assays hydroxymethylbilane synthase (Hs_HMBS_1_SG), S1P_1_ (Hs_S1PR1_1_SG), S1P_2_ (Hs_S1PR2_1_SG), S1P_3_ (Hs_S1PR3_1_SG), S1P_5_ (Hs_S1PR5_1_SG), ABCA1 (Hs_ABCA1_1_SG), SPT (Hs_SPTLC1_1_SG, Hs_SPTLC2_1_SG, and Hs-SPTLC3_2_SG), were from Qiagen (Hilden, Germany). The siRNAs targeting SPTLC1 (Hs_SPTLC1_12 and Hs_SPTLC1_13), SPTLC2 (Hs_SPTLC2_1 and Hs_SPTLC2_6), SPTLC3 (Hs_SPTLC3_2 and Hs_SPTLC3_6), S1P_1_ (Hs_S1PR1_5), S1P_2_ (Hs_S1PR2_6), S1P_3_ (Hs_S1PR3_6), S1R_5_ (Hs_S1PR5_6), and ABCA1 (Hs_ABCA1_2 and Hs_ABCA1_5) were also from Qiagen. Non-targeting siRNA (‘siScr’) was from Dharmacon (Thermo Scientific, MA, USA). Guava ViaCount Reagent was from Merck Millipore (Darmstadt, Germany).

### 2.2 Cells and culture conditions

The human glioma cell lines U87MG and U251MG were purchased from CLS-Cell line services, Germany or ATTC, LGC Standards, Germany and maintained in DMEM/high glucose supplemented with 10% fetal calf serum (FCS) and 2% penicillin/streptomycin at 37 °C under 5% CO_2_. Establishment and culture of GM133 cells has been previously described in detail [33]. Primary GBM2 were established from glioblastoma multiforme tissue obtained during surgery and diagnosed according to the WHO classification. The p53 status of GBM2 cells was sequenced and described recently [34]. The protocol was approved by the local ethical review boards. Cells were cultured in DMEM/high glucose supplemented with 10% FCS and 2% penicillin/streptomycin at 37 °C under 5% CO_2_ and used up to passage 10.

### 2.3 RNA interference

Cells were seeded at 50,000 cells per well into 12 well plates and grown for 24 h. Transfection of siRNA (20 nM) was performed with Oligofectamine according to the manufacturer's suggestions (Invitrogen). Untreated cells (control) and cells transfected with Oligofectamine alone (mock) or scrambled siRNA (siScr) were used as controls.

### 2.4 Western blot analysis

For immunoblotting, protein concentration of whole cell extracts and mouse brain lysates was measured using the Bradford protein assay. Equal protein aliquots were loaded, separated on SDS-PAGE under reducing conditions, transferred to PVDF membranes and probed with specific primary antibodies as described in Materials. Immunoreactive bands were detected with HRP-conjugated secondary antibodies. Protein expression was visualized using ECL reagents. Membranes were stripped and re-probed for β-actin (1:5000).

### 2.5 Proliferation

Cells were seeded at 50,000 per well into 12 well culture plates, grown for 24 h and transfected with specific siRNA (20 nM). At the indicated time points cells were trypsinized and the cell number was determined using a Casy Cell Counter or Guava ViaCount Assay on Guava EasyCyte 8 (Millipore).

### 2.6 Treatment with pharmacological inhibitors

Cells (50,000) were plated into 12 well and grown for 24 h. Then, myriocin (in DMSO; 1 and 5 μM; Ref. [35]) and DMSO as vehicle control (≤0.05%) was added daily in medium without serum. For treatment with glyburide (200 μM; Ref. [30]) and DIDS (400 μM; Ref. [36]), U87MG cells were seeded at 100,000 cells per well into 12 well culture plates and allowed to attach overnight. The cells were serum starved for 24 h and treated with glyburide or DIDS, and the corresponding vehicle controls as indicated. Cell numbers were determined as described above.

### 2.7 SL analysis

Three days post-SPTLC1 silencing or overnight treatment with myriocin (1 μM serum-free) cells were harvested and sonicated in cold phosphate buffered saline (PBS; pH 7.4). Samples were mixed with three volumes of chloroform/methanol (2:1; v/v) with Cer17:0 and SM17:0 as internal standards (Avanti Polar Lipids, Alabaster, USA). For SL analysis lipid extracts were subjected to a mild alkaline hydrolysis step by adding 400 μl 1 N NaOH in methanol/CHCl_3_/H_2_O (10:5:1; v/v/v) and incubated for 45 min at room temperature. Samples were then neutralized by adding 150 μl 1 M acetic acid and 400 μl 0,5 M EDTA. After adding 1 ml CHCl_3_, samples were vortexed, centrifuged, and the upper aqueous layer was removed. Then, 700 μl H_2_O were added and after vortexing the aqueous phase was removed again and the lower organic layer was dried under N_2_. Lipids were separated on a C-18 UPLC-column and analyzed with a QTOF-MS system as described [37]. Data analysis was performed using the Lipid Data Analyzer Software [38].

### 2.8 Quantification of S1P

For S1P measurements cells were seeded in 21 cm^2^ Petri dishes. When approximately 80% confluent, cells were treated with myriocin (1 μM, overnight in serum) or glyburide (500 μM for 10 h and 200 μM for 14 h, serum-free). DMSO was used as vehicle control. Quantification of intracellular S1P was performed with a commercially available ELISA kit (Echelon Biosciences, UT, US) according to the manufacturer's suggestions. For quantitative analysis of samples a nonlinear regression model (as suggested by the manufacturer) was used and data were normalized to protein content.

#### 2.8.1 Annexin V/propidium iodide staining

Cells were transfected with siRNA or treated with 1 μM myriocin as described above. On day five cells were harvested and stained using the FITC Annexin V Apoptosis Detection Kit 1 (BD Biosciences, USA) as recommended by the manufacturer. Briefly, cells were washed in cold PBS and incubated for 15 min at room temperature in the dark in 100 μl of 1× binding buffer containing 5 μl of Annexin V FITC and 5 μl of propidium iodide (PI). Flow cytometric analyses were performed on Guava EasyCyte 8 (Millipore, Billerica, MA, USA) and analyzed using ModFit (Verity Software House). To set up fluorescent compensation and gating for the detection of early and late apoptosis, unstained and single stained positive controls treated with staurosporine (Enzo Life Sciences, Switzerland; 1 μM, 4 h) or H_2_O_2_ (3 mM, 4 h) were used.

### 2.9 RT-qPCR

Cells were transfected with siRNA, lysed, and RNA extracts were collected at the indicated time points. Total RNA was isolated using the RNeasy Plus Kit. Aliquots of three μg of total RNA were reverse transcribed using SuperScript III Reverse Transcriptase and random hexamer primers according to the manufacturer's instructions (Invitrogen). RT-qPCR was performed with an Applied Biosystems 7900HT Fast Real Time PCR System, the QuantiFast SYBR Green PCR kit and QuantiTect Primer Assays. HMBS or hypoxanthine phosphoribosyltransferase (HPRT) was used as housekeeping gene.

#### 2.9.1 S1P-enrichment of high density lipoprotein (HDL) or BSA

Human HDL (4 mg protein/ml; isolated as described [39]) or fatty-acid free BSA (4 mg/ml) were incubated with S1P (50 μg) on a rotating wheel for 4 h at 37 °C. S1P content was determined as described above.

### 2.10 S1P treatment

U87MG cells were seeded at 40,000 cells per well into 12 well culture plates and allowed to attach overnight. The cells were serum starved for 24 h and received BSA-bound (S1P-BSA) or HDL-bound S1P (S1P-HDL) at the indicated concentrations. S1P-BSA or S1P-HDL was added every 24 h to overcome short half-life in the cellular supernatant [40]. Six days after plating cells were trypsinized and the cell number was determined with a Casy Cell Counter.

### 2.11 Statistical analyses

Data are presented as mean ± SD. Either Student's *t*-test or ANOVA was used for analysis of statistical significance (using the GraphPad Prism package). All values of *p* < 0.05 were considered significant. Statistical significance of differences in mRNA expression levels was analyzed using the relative expression software tool (REST^©^, http://www.gene-quantification.de/rest.html) using a pair-wise fixed reallocation test [41].

## 3 Results

### 3.1 Silencing of SPTLC1 or inhibition of SPT impairs cell growth, alters cellular SL profiles, and induces apoptosis

**Fig. 1:**
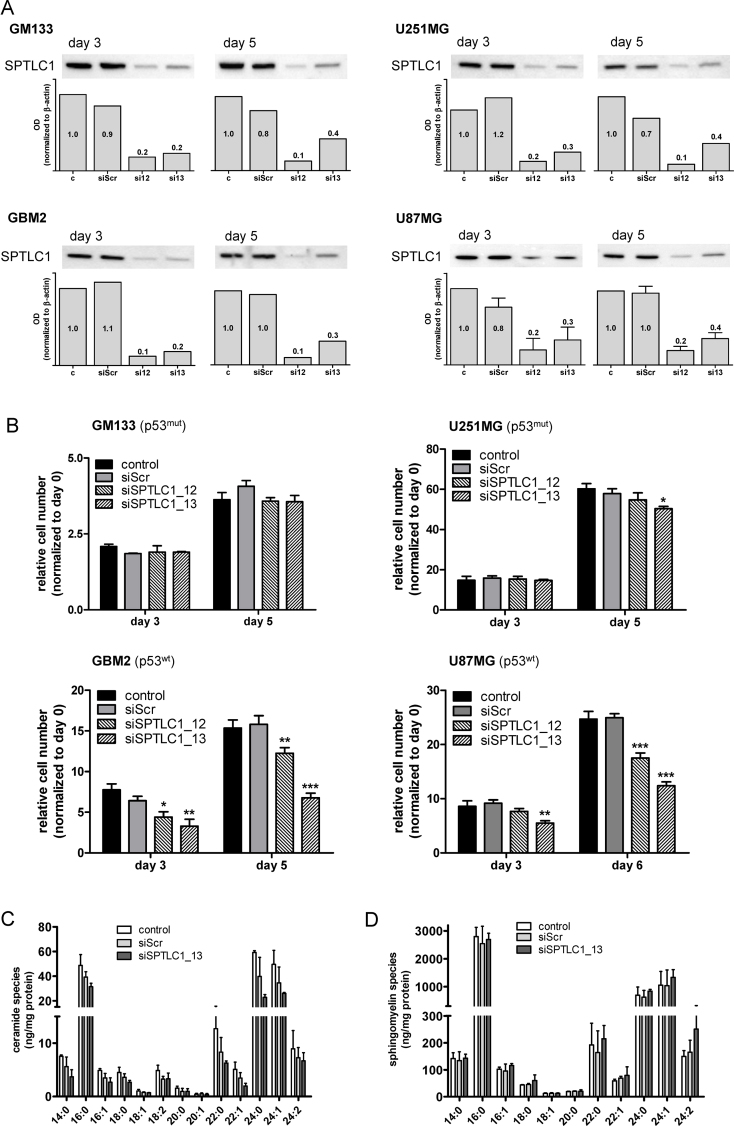
Impact of SPTLC1 silencing on glioma cell proliferation and sphingolipid profiles. (A) At days 3 and 5 post-silencing (siSPTLC1_12 and _13) SPTLC1 expression was analyzed by Western blotting in p53^mut^ (GM133 and U251MG) and p53^wt^ (GBM2 and U87MG) cells. Untreated cells (c) and cells transfected with scrambled siRNA (siScr) were used as controls. The bar graph represents SPTLC1 optical density (OD) normalized to actin (two and four (U87MG) independent experiments). The SPTLC1/actin ratio of untreated cells (c) was set to 1 and the mean OD ratios are displayed numerically. One representative blot is shown as inset. (B) Following knockdown (siSPTLC1_12 and _13) of SPTLC1 cell numbers were counted at the indicated time points. Results shown represent mean ± SD from triplicate experiments. Untreated cells (control) and cells transfected with scrambled siRNA (siScr) were used as controls. *p < 0.05, **p < 0.01, ***p < 0.001 compared to siScr (one-way ANOVA). (C) Ceramide and (D) sphingomyelin composition was analyzed in U87MG cells and quantitated by LC–MS/MS analysis in control cells or cells transfected with siScr or siSPTLC1_13. Lipids were extracted, separated on a C-18 UPLC-column, and analyzed with a QTOF-MS system. Data analysis was performed using the Lipid Data Analyzer Software. Results represent mean ± SD from triplicate experiments. Cer and SM species are displayed on basis of their acyl chain composition.

To elucidate the contribution of de novo SL synthesis to cell proliferation, SPTLC1, a catalytic long chain base subunit of SPT catalyzing the first and committed step of SL synthesis, was silenced in a panel of glioma cells. To get an indication whether the p53 status determines the outcome of SPTLC1 silencing on cell proliferation we used three established glioma cell lines (U87MG, U251, and GM133) and one low passage culture (GBM2) that was established by our group; of these cells, GM133 and U251 are p53^mut^, while GBM2 and U87MG are p53^wt^ [34]. Despite efficient knockdown of SPTLC1 protein levels in response to RNAi (Fig. 1A; decrease of immunoreactive SPTLC1 by 60–90%; bar graphs) only GBM2 and U87MG cells responded with reduced proliferation (Fig. 1B). In both p53^wt^ glioma cultures proliferation was reduced to 45 and 50% of controls (Fig. 1B; GBM2 and U87MG, respectively).

Next, cellular Cer and SM composition was quantitated in SPTLC1-silenced U87MG cells. LC–MS analyses revealed a total Cer and SM content of 210 and 5269 ng/mg cell protein in untreated U87MG glioma cells (Fig. 1C and D). The C16:0, 22:0, 24:0, and 24:1 species contributed the majority of Cer (81%) and SM (90%). In response to SPTLC1 silencing, total Cer levels decreased by 48% in comparison to controls (Fig. 1C; 109 vs. 210 ng/mg cell protein) and by 36% in comparison to siScr (109 vs. 170 ng/mg cell protein). SM concentrations (except the C24:2 species) were unaffected by SPTLC1 silencing (Fig. 1D).

**Fig. 2:**
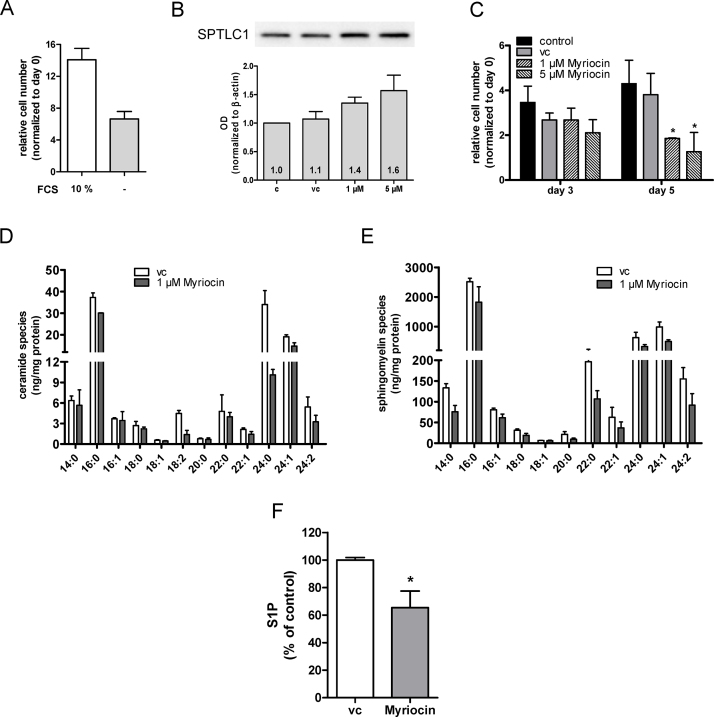
Impact of the SPT inhibitor myriocin on U87MG cell proliferation and sphingolipid profiles. (A) U87MG cell proliferation in the presence (10%) or the absence of FCS. Cell numbers were counted 5 days post plating. (B) SPTLC1 expression in control (c), vehicle (vc), and myriocin-treated cells was analyzed by Western blotting. The bar graph represents SPTLC1 band intensity normalized to actin (four independent experiments). The SPTLC1/actin ratio of untreated cells (c) was set to 1 and the mean OD ratios are displayed numerically. One representative blot is shown as inset. (C) Untreated cells (control) or cells treated with vehicle (DMSO; vc) or myriocin under serum-free conditions were counted at the indicated times. Results shown represent mean ± SD from triplicate experiments. *p < 0.05 compared to vehicle control (one-way ANOVA). (D) Ceramide and (E) sphingomyelin composition was quantitated by LC–MS analysis in vehicle (vc) or myriocin-treated cells as described in Fig. 1C and D. Results represent mean ± SD from triplicate experiments. Cer and SM species are displayed on basis of their acyl chain composition. (F) Cells were incubated overnight in medium containing DMSO as vehicle control (vc) or myriocin (1 μM). S1P levels of cell lysates were determined using a competitive ELISA. Data are presented as mean ± SEM (two independent experiments performed in duplicates). Unpaired Student's *t*-test was used for analysis of statistical significance. *p < 0.05 compared to vehicle.

As alternative approach SPT activity was inhibited with myriocin [42]. These experiments were performed in serum-free medium. Under these conditions U87MG cells still proliferate although approx. twofold slower than in serum-containing medium (Fig. 2A). Myriocin tended to increase SPTLC1 protein levels (up to 1.6-fold at 5 μM; Fig. 2B). Pharmacological inhibition of SPT reduced cell numbers by 55 and 75% at day 5 (1 and 5 μM myriocin; Fig. 2C). Myriocin treatment led to statistically significant reduction of the C16:0, 18:1, 18:2, 22:1, 24:0, and 24:1 Cer species (Fig. 2D; 73 vs. 121 ng/mg cell protein) and also decreased the content of several SM species (Fig. 2E; total SM content = 3062 vs. 4706 ng/mg cell protein; myriocin- vs. vehicle-treated cells). Finally, myriocin treatment induced a significant decrease of intracellular S1P concentrations by 35% (Fig. 2F).

**Fig. 3:**
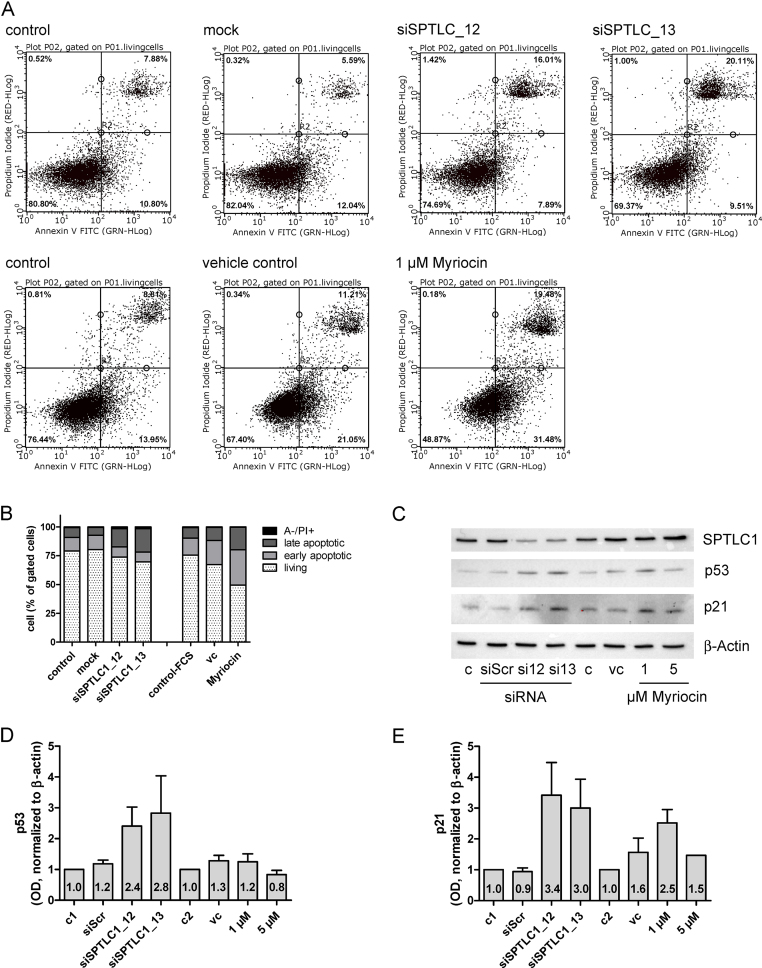
Genetic and pharmacological inhibition of SPT induces apoptosis. (A) U87MG cells were either untransfected or transfected with Oligofectamine (mock) or SPTLC1-targeting siRNAs (day 5; upper panel). Control cells, vehicle- and myriocin-treated cells (1 μM, day 5) are shown in the lower panel. The cells were trypsinized, stained with Annexin V-FITC (A) and propidium iodide (PI) and analyzed by flow cytometry. Cells in the lower right quadrant represent A+/PI− early apoptotic cells and these in the upper right quadrant (A+/PI+) represent late apoptotic cells. To set up fluorescent compensation and gating, unstained and single stained positive controls (1 μM staurosporine or 3 mM H_2_O_2_, 4 h) were used. Untreated (control), mock transfected (mock), SPTLC1-silenced, DMSO (vehicle control) and myriocin (1 μM)-treated cells were analyzed. (B) The bar graph shows the proportion of A−/PI− (living), A+/PI− (early apoptotic), A+/PI+ (late apoptotic), and A−/PI+ cells from two independent experiments performed in triplicates. (C) The effects of SPT inhibition using RNAi (siSPTLC1_12, _13) and myriocin on SPTLC1, p53, p21, and β-actin expression were analyzed by Western blotting. Following densitometric Western blot analysis p53 (D) and p21 (E) band intensities were normalized to actin (four independent experiments). The p53- and p21/actin ratio of untreated cells (c1 = silencing-, c2 = myriocin-controls) was set to 1 and the mean OD ratios are displayed numerically. One representative blot is shown as inset.

To study whether reduced cell proliferation due to RNAi or myriocin is accompanied by apoptosis, Annexin V-FITC (A)/PI staining was performed (Fig. 3). Under non-treated conditions the number of early and late apoptotic cells was 11 and 8%, respectively (Fig. 3A; upper panel). Mock transfection with Oligofectamine was without effect on cell viability. Silencing of SPTLC1 increased the percentage of late apoptotic cells (16 and 20%; siSPTLC_12 and _13, respectively; Fig. 3A, upper panel). In comparison, 31% of myriocin-treated cells were early and 19% late apoptotic (Fig. 3A; lower panel). Also DMSO used as vehicle control increased the percentage of early and late apoptotic cells (21 and 11%, respectively). Fig. 3B summarizes the percentage of living, early and late apoptotic, and A−/PI+ cells. To get an indication about underlying pathways Western blot analysis of silenced and myriocin-treated cells was performed. Results of these experiments (Fig. 3C) revealed upregulation of p53 and p21 in silenced (and to a lower extent in myriocin-treated) cells, indicating a pro-apoptotic role of these tumor suppressors when de novo SL synthesis is inhibited. Statistical evaluation of p53 and p21 band intensities is shown in Fig. 3D and E.

**Fig. 4:**
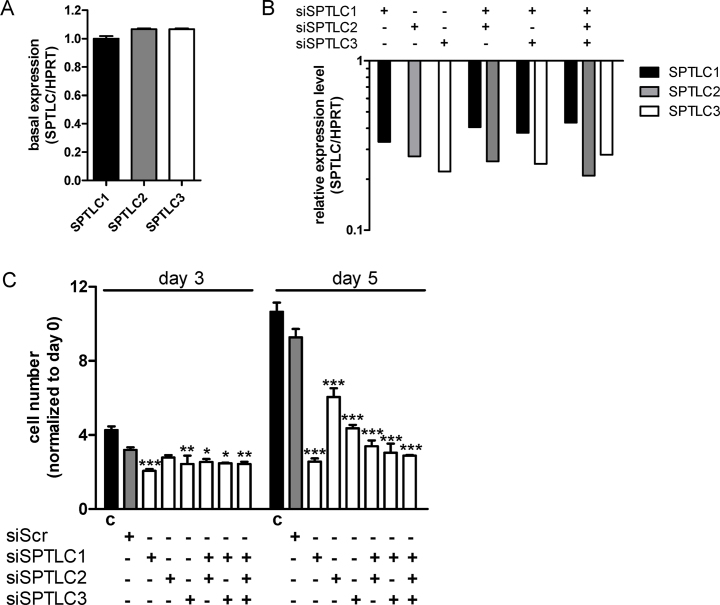
Characterization of SPTLC family member expression and silencing effects on U87MG proliferation. (A) SPTLC subunit expression by U87MG cells grown under standard conditions was analyzed by RT-qPCR. Target gene expression was normalized to HPRT. Gene expression ratios were calculated by REST as described in Materials and Methods. SPTLC1 expression was arbitrarily set to 1. (B) At day 3 post-silencing SPTLC1, -2, and -3 RNA levels were quantitated by RT-qPCR. Target gene expression in silenced cells was normalized to target gene expression in mock transfected cells. Gene expression ratios were calculated by REST as described in Materials and Methods. (C) Following knockdown of the indicated SPTLC family members cell numbers were counted at the indicated time points. Results shown represent mean ± SD from triplicate experiments. Untreated cells (control, ‘c’) and cells transfected with scrambled siRNA (siScr) were used as controls. *p < 0.05, **p < 0.01, ***p < 0.001 compared to siScr (one-way ANOVA).

The SPT subunits SPTLC1, -2, and -3 are approx. equally expressed by U87MG cells on mRNA level (Fig. 4A). To examine the contribution of the remaining SPT subunits on cell proliferation we silenced also SPTLC2 and -3 and performed a co-silencing approach. Silencing efficacy (determined by qPCR) was between 57 and 79% (Fig. 4B). Also the double (SPTLC1/2 and SPTLC1/3) and triple (SPTLC1/2/3) silencing approaches resulted in efficient knockdown. However, silencing of the other SPT subunits (SPTLC2 and -3) either alone or in combination (SPTLC1/2; SPTLC1/3, and SPTLC1/2/3) did not further decrease cell numbers as compared to single SPTLC1 knockdown (Fig. 4C).

### 3.2 The contribution of S1P receptors to U87MG proliferation

**Fig. 5:**
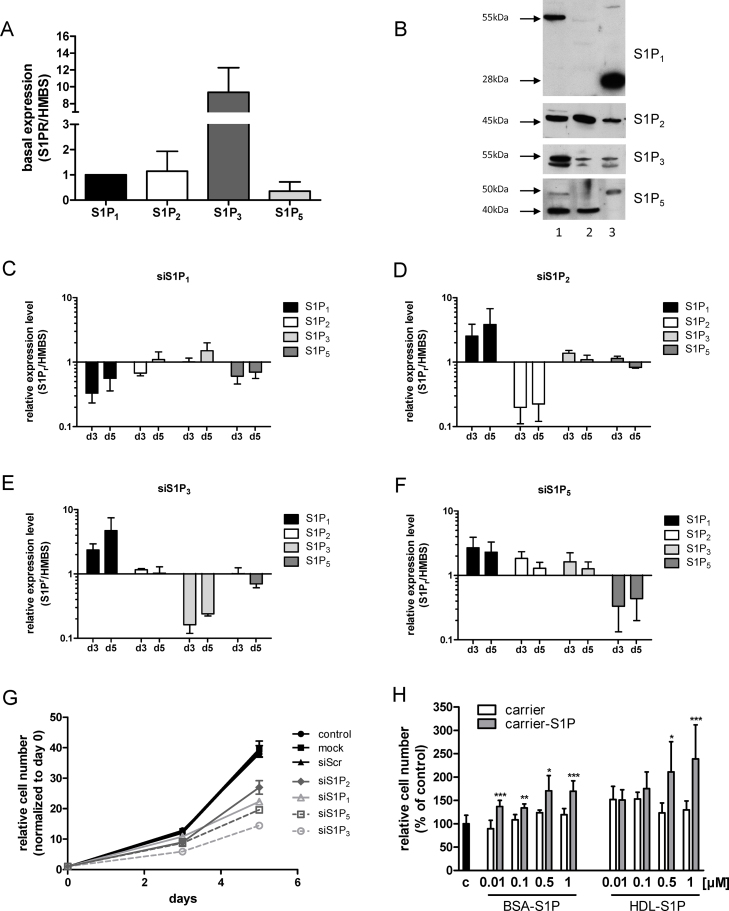
S1P receptor expression and silencing. (A) S1P receptor subtype expression by U87MG cells grown under standard conditions was analyzed by RT-qPCR. Target gene expression was normalized to HMBS. Gene expression ratios were calculated by REST as described in Materials and Methods. S1P_1_ expression was arbitrarily set to 1. (B) S1P receptor subtype expression by U87MG cells (lane 1), HeLa cells (lane 2) and mouse brain protein lysates (lane 3) was analyzed by Western blotting. Molecular masses are indicated. (C–F) Effect of S1P receptor knockdown on mRNA levels of targeted and non-targeted receptor family members. Receptor silencing was performed with siRNA constructs showing highest silencing efficacy in a pre-screen. Relative gene expression of target genes (as analyzed by real time qPCR) at days 3 and 5 (d3, d5) post silencing is presented in relation to HMBS. Results represent mean ± SD from three independent experiments. (G) Effect of S1P receptor silencing on cell proliferation. Individual S1P receptors were silenced as described above. Cells were counted at days 3 and 5 post silencing. Untreated cells (control) and cells transfected with Oligofectamine (mock) or scrambled siRNA (siScr) were used as controls. Results are cell numbers normalized to day zero and represent mean ± SD from triplicate determinations. (H) Effects of exogenously added S1P on cell proliferation. Cells were grown in the absence (c) or the presence of the indicated S1P concentrations complexed either to BSA (BSA-S1P) or HDL (HDL-S1P). After five days cells were washed, trypsinized and counted. Results represent mean ± SD from two independent experiments done in triplicates. Vehicle control (‘carrier’) consisting of BSA or HDL (‘carrier-S1P’). *p < 0.05, **p < 0.01, ***p < 0.001 compared to the corresponding vehicle control (unpaired Student's *t*-test).

Having established that silencing or inhibition of SPT impacts on SL, S1P content, and cell viability the role of individual S1P receptors that transmit S1P-dependent growth signals was investigated. Under basal conditions highest mRNA levels were observed for S1P_3_ (S1P_3_/HMBS = 9), followed by S1P_2_ and S1P_1_; the latter was arbitrarily set to 1 (Fig. 5A). Lowest expression was found for S1P_5_ while no S1P_4_ could be detected on RNA level. The molecular masses (Western blotting) of S1P receptors present on U87MG cells were 55 (S1P_1_), 45 (S1P_2_), 52 and 55 (S1P_3_), 40 and 50 (S1P_5_) kDa (Fig. 5B, lane 1). The pattern of immunoreactive bands for S1P receptors in HeLa cells (Fig. 5B, lane 2), used as controls, was comparable to U87MG. In contrast immunoreactive bands in mouse brain protein lysates (Fig. 5B, lane 3) were detected at 28 and 50 kDa using S1P_1_ and S1P_5_ antibodies, respectively.

To get an indication about the quantitative contribution of individual S1P receptors to U87MG cell proliferation we used RNAi. This approach was applied to clarify silencing efficacies and potential off-target or counter-regulatory effects on non-targeted S1P receptors on mRNA level (Fig. 5C–F). In general, silencing was efficient and mRNA levels of targeted receptors were downregulated between 70 and 90%. Silencing of S1P_1_ was without pronounced effects on expression levels of non-targeted receptors (Fig. 5C). In contrast, silencing of S1P_2_, S1P_3_, and S1P_5_ was paralleled by up-regulation of S1P_1_ between two- and fivefold (Fig. 5D–F). Silencing of S1P_5_ (Fig. 5F) induced transcriptional upregulation of S1P_2_ and S1P_3_ at day 3 post silencing.

Next, effects of S1P receptor subtype silencing on U87MG proliferation was studied. These data (Fig. 5G) showed that all S1P receptors impact on U87MG proliferation, though to different degrees: S1P_1_ silencing induced a reduction in cell numbers (day 5 post silencing) by 45%. S1P_2_ knockdown resulted in growth inhibition by 30%. Knockdown of S1P_3_ and S1P_5_ reduced cell numbers by 63 and 50%. In light of counter-regulations identified in Fig. 5D–F it was not possible to unambiguously identify the receptor with the highest impact on U87MG proliferation.

The majority of physiologically active S1P (that promotes S1P receptor-mediated downstream signaling) is transported in association with albumin and/or HDL [43]. Therefore the effects of these physiological S1P carriers on cell proliferation were studied. Exogenous S1P at physiologically relevant concentrations (10 nM to 1 μM; Ref. [44]) led to increased U87MG cell proliferation (Fig. 5H). While BSA-complexed S1P increased cell numbers by a maximum of 1.4-fold, HDL-associated S1P enhanced proliferation rates by a maximum of 1.8-fold in comparison to vehicle-treated cells.

### 3.3 ABCA1 is a potential contributor to S1P inside out signaling

**Fig. 6:**
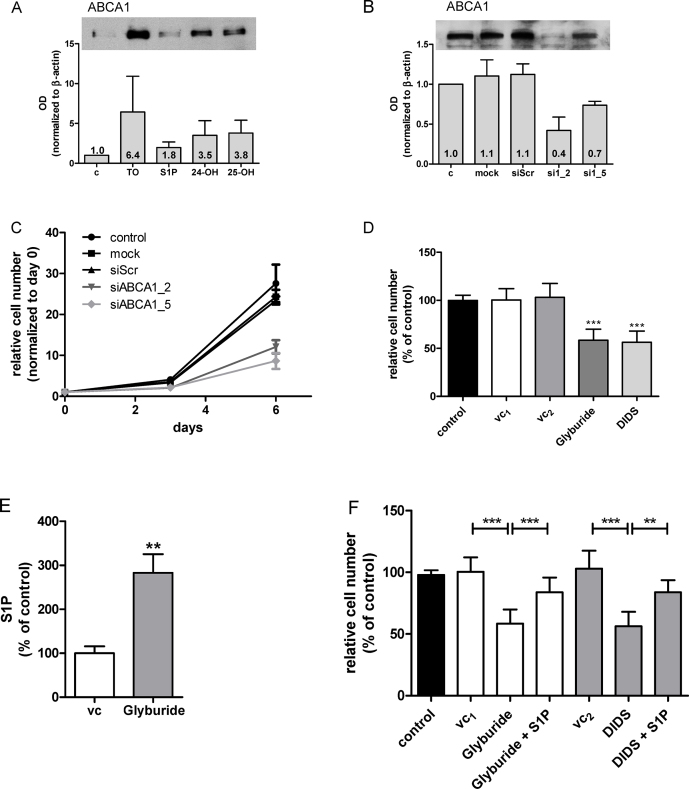
Silencing and pharmacological inhibition of ABCA1 reduces U87MG proliferation and increases intracellular S1P concentrations. (A) Expression of ABCA1 was determined in cells cultured in the absence (‘c’) or the presence of a synthetic LXR ligand (TO901317; 10 μM; ‘TO’), S1P (1 μM), or natural LXR ligands (24-OHcholesterol; ‘24-OH’; 1 μM; 25-OHcholesterol; ‘25-OH’; 1 μM). After 3 days cellular lysates were analyzed by Western blotting. The bar graph represents ABCA1 band intensity normalized to actin (three independent experiments). The ABCA1/actin ratio of untreated cells (c) was set to 1 and the mean OD ratios are displayed numerically. One representative blot is shown as inset. (B) ABCA1 expression was analyzed in untreated (c) cells and cells subjected to transfection with Oligofectamine (mock), scrambled siRNA (siScr), and two ABCA1 siRNAs (si1_2 and si1_5). The bar graph represents ABCA1 band intensity normalized to actin (three independent experiments) and mean OD are displayed numerically. The ABCA1/actin ratio of untreated cells (c) was set to 1 and the mean OD ratios are displayed numerically. One representative blot is shown as inset. (C) Untreated cells (control) and cells subjected to transfection with Oligofectamine (mock), scrambled siRNA (siScr), and two ABCA1 siRNAs (si1_2 and si1_5) were harvested at days 3 and 6 post silencing and counted. Results are cell numbers normalized to day zero and represent mean ± SD of triplicate dishes. (D) ABCA1 was pharmacologically inhibited with glyburide (200 μM, in DMSO) or DIDS (400 μM in PBS). After three days cells were washed, trypsinized and counted. Results represent mean ± SD from triplicate dishes. ***p < 0.001 compared to vehicle controls (control = untreated, vc_1_ = DMSO, vc_2_ = PBS). (E) Cells were incubated for 24 h in medium containing DMSO (vc) in the absence or the presence of glyburide (500 μM for 10 h followed by 200 μM for 14 h). S1P concentrations of cellular lysates were determined using a competitive ELISA. Data are presented as mean ± SEM (two independent experiments performed in triplicate). Unpaired Student's *t*-test was used for analysis of statistical significance. **p < 0.01 compared to vehicle control. 100% of S1P corresponds to 221 pmol/mg cell protein. (F) Cells were incubated for 24 h in the absence (control) or the presence of glyburide (200 μM), DIDS (400 μM), or the corresponding vehicles (DMSO or PBS, vc_1_ and vc_2_, respectively). Where indicated, cells received (in addition to glyburide or DIDS) S1P (1 μM). After three days cells were washed, trypsinized and counted. Results represent mean ± SD from triplicate dishes. **p < 0.01, ***p < 0.001 compared to the corresponding vehicle control (one-way ANOVA).

Finally we sought evidence whether ABCA1 could contribute to S1P efflux in U87MG cells, a mechanism reported for non-transformed astrocytes [31]. U87MG cells express ABCA1 and addition of exogenous S1P slightly increased ABCA1 levels (Fig. 6A). In contrast, a synthetic liverXreceptor (LXR) ligand (TO901317) and the natural LXR ligands 24- and 25-OHcholesterol enhanced ABCA1 expression 3.5–6.4-fold (Fig. 6A). Silencing of ABCA1 reduced protein levels between 30 and 60% (Fig. 6B) and inhibited cell proliferation by 50 and 65% (Fig. 6C). Comparable results were obtained with pharmacological ABCA1 antagonists (glyburide and DIDS) that reduced cell numbers by approx. 40% (Fig. 6D). Cell lysates from glyburide-treated cells revealed S1P concentrations 2.7-fold higher as compared to vehicle-treated cells (Fig. 6E) indicating reduced S1P efflux in response to pharmacological ABCA1 antagonism. Addition of exogenous S1P reverted the proliferative block mediated by glyburide and DIDS (Fig. 6F). These findings suggest that genetic or pharmacological interference with S1P efflux holds promise to inhibit cancer cell proliferation.

## 4 Discussion

The present study aimed to interfere with key synthetic and signaling nodes of SL turnover in U87MG glioma cells. The present in vitro data suggest that interference with de novo SL synthesis, signaling via S1P receptors, or efflux of S1P (ABCA1) attenuates U87MG glioma cell proliferation.

To investigate the impact of de novo SL biosynthesis on proliferative behavior we have modulated the expression or activity of a key enzyme involved in this pathway. SPTLC1 and the heterodimer formed with SPTLC2 or SPTLC3 constitutes the catalytic core of the enzyme. RNAi of SPTLC1 was efficient and downregulated proliferation of GBM2 and U87MG glioma cells that express p53^wt^, but was without effect on GM133 and U251MG, that express p53^mut^ (Fig. 1). All of the remaining experiments were performed with p53^wt^ U87MG cells. Inhibition of SPT with myriocin reduced cell numbers by 40–70% (Fig. 2). This is comparable to what was reported for myriocin-induced cell cycle arrest in lymphocytes: In that study [45] myriocin reduced proliferation by approx. 50%.

We here show that RNAi of SPTLC1 reduced cellular Cer content by 37% with the quantitatively most pronounced changes seen in C16:0, C24:0, and C24:1 while the SM composition was almost unaffected (Fig. 1C and D). Surprisingly, transfection with siScr also diminished the cellular Cer content (Fig. 1C); currently the reason for this observation is not clear. In response to myriocin the quantitatively most pronounced decrease was observed for C24:0 Cer (Fig. 2D). In contrast to the siRNA approach myriocin induced also a decline in most SM species (Fig. 2E). Pharmacological manipulation of SL metabolism in tumor cells holds promise as new therapeutic modality in cancer. However, tuning the SL rheostat in the direction of Cer synthesis might be insufficient since some cancer types like endometrial cancer [46], breast tumor biopsies [47], or murine xenografts [48] have higher Cer content as compared to non-transformed tissue.

Therefore we decided to inhibit de novo SL biosynthesis to interfere with glioma cell proliferation. Feasibility of such an approach was demonstrated in melanoma, where myriocin treatment induced growth arrest in vitro [49] and suppressed tumor growth in a murine melanoma model via p53- and p21-dependent pathways [50] as observed during the present study (Fig. 3C–E). This might be of importance for future studies since SPTLC1 silencing was without effect on GBM cell proliferation that express p53^mut^ (Fig. 1B). In liver cells silencing of the SPTLC subunits 1–3 reduced Cer levels by approx. 35% [51], comparable to what we have found in U87MG cells (Fig. 1C). Of note, SPTLC1 silencing had the most pronounced effects on cell proliferation during the present study (Fig. 4). SPTLC silencing impacted global gene expression with upregulation of negative regulators of biosynthetic processes demonstrating the possibility of energy deprivation in response to decreased Cer synthesis [51]. In the intestine, conditional knockout of SPTLC2 induced necrotic lesions at the bases of villi and crypts, indicating the requirement for de novo Cer biosynthesis via SPT during proliferation [52]. Finally, Cer is the central metabolite of SL turnover and it is conceivable that membrane synthesis, architecture, and function could be profoundly disturbed under Cer-depleted conditions [53].

There is consensus that Cer species with specific acyl chain length may have unique cellular functions [54]. In terms of species specificity it was shown that C18:0 Cer induces cancer cell death and decreases tumor growth [55]. In contrast, C16:0 (which is increased in glioma; Ref. [19]) and C24:0 Cer can increase cancer cell proliferation and protect from cell death [56]. Knockdown of CerS2 (the Cer synthase member catalyzing C24 Cer formation) in HeLa cells resulted in nearly complete absence of C22 and C24 Cer species and significantly enhanced sensitivity toward cisplatin-induced apoptosis [57]. Thus, a reduction in C16:0 and/or C24:0 Cer as observed during the present study (Figs. 1C and 2D) could account for reduced cell viability of U87MG cells where SPT function was genetically or pharmacologically inhibited. However, it remains to be determined whether changes in the composition of SL chain length affect apoptosis signaling directly or indirectly, e.g. via changes in membrane properties such as microdomain formation. Another possibility that was not addressed here is reduced formation of pro-proliferative Cer-1-P [58] as a consequence of substrate shortage in SPTLC-silenced or myriocin-treated glioma cells. Finally, ceramides are capable of inducing either survival or lethal autophagy (reviewed in [54]), however, these pathways were not followed up during the present study.

Pharmacological inhibition of SPT with myriocin reduced the intracellular S1P concentrations (Fig. 2F). S1P-dependent signaling networks are elicited by the corresponding S1P receptors. RT-qPCR and Western blot analysis revealed expression of S1P_1–3,5_ on U87MG cells (Fig. 5). This is in line with S1P receptor expression patterns identified in human GBM tissue [20,21]. Van Brocklyn and Young [24] reported that S1P_1–3_ contribute to U-118MG and U-373MG glioma cell proliferation, with S1P_1_ being the most important regulator. These authors [24] reported that S1P_3_ and S1P_1_ mediate glioma cell migration and invasion. High expression of S1P_1_ correlates with high invasive potential of CD133^+^ GBM cells [15,16]. S1P_2_ inhibits GBM cell migration [22–24] but upregulates their invasive potential [24]. Our finding that S1P_1_ was consistently upregulated in response to S1P_2_, S1P_3_ and S1P_5_ silencing (Fig. 5C–F) deserves attention. Although the ability of S1P receptors to homo- and heterodimerize is established [59,60], it is currently not clear whether functional loss of one binding partner could induce transcriptional regulation of the other.

Finally we showed that silencing or inhibition of ABCA1 decreased U87MG proliferation. ABCA1 expression in U87MG cells was potently induced by 25-OHcholesterol (Fig. 6A), a natural LXR ligand and oxysterol synthesized and secreted by glioma cells [61]. This supports the notion that oxysterol synthesis provides an autocrine signal that enhances ABCA1 expression thereby increasing S1P export from glioma cells. Of note, glyburide induced accumulation of intracellular S1P and exogenously added S1P partially restored cell proliferation in the presence of ABCA1 inhibitors (Fig. 6F). These findings would be compatible with reduced ABCA1-dependent efflux of this bioactive lipid mediator in U87MG glioma cells. However, the ABCA1 transporter actively transports also other lipids, namely cholesterol, phosphatidylcholine, phosphatidylserine, and SM [62]. Therefore pharmacological antagonism or silencing of ABCA1 could result in chronic exposure to high concentrations of intracellular cholesterol that compromise (U87MG) cell viability. The fact that exogenously added S1P to DIDS- and glyburide-treated cells only partially restored cell proliferation (Fig. 6F) could substantiate the existence of such a mechanism.

In addition to other S1P transporters [63], ABCA1 plays a critical role in S1P efflux to newly synthesized HDL-like particles in astrocytes [31]. Noteworthy, in a patient cohort of type 2 diabetes mellitus glyburide treatment was associated with reduced cancer risk [64]. In animal models glyburide (in combination with CoCl_2_) inhibited the growth of breast cancer xenografts [65]. In vitro, glyburide was shown to induce G0/G1 arrest in breast cancer cells [66] and to suppress invasive properties of ovarian carcinoma cells [67]. Finally, high expression of ABCA1 (and other members of the ABCA family) correlates with reduced survival in serous ovarian cancer patients and siRNA-mediated suppression of ABCA1 inhibited ovarian cancer cell growth and migration in vitro [68].

Overall, data presented here identify de novo SL biosynthesis, as well as S1P outside-in and inside-out signaling as attractive pathways to interfere with proliferation in GBM cells expressing p53^wt^. However, the clinical situation might be more complex, especially in terms of altered SL synthesis/turnover in response to chemo- and/or radiation therapy.
